# Mapping ecological paradigms in educational psychology: a decade of scholarship in *Frontiers in Psychology* (2013–2025)

**DOI:** 10.3389/fpsyg.2026.1793016

**Published:** 2026-04-13

**Authors:** Peiru Tong, Irene Shidong An, Samantha Zhan Xu

**Affiliations:** 1 School of International Education, Wuhan University, Wuhan, Hubei, China; 2 Discipline of Chinese Studies, School of Languages and Cultures, The University of Sydney, Sydney, NSW, Australia

**Keywords:** Bronfenbrenner, ecological perspective, ecological systems theory, bioecological theory, ecology, ecosystem, educational psychology, human development

## Abstract

**Introduction:**

Educational psychology has increasingly drawn on ecological perspectives to address the complexity of learning and development across contexts. This review synthesizes educational psychology studies published in Frontiers in Psychology between 2013 and 2025 that explicitly invoke ecological perspectives. The study investigates three main research questions: (1) What educational contexts, populations, research topics, and methodological approaches characterize ecological research in educational psychology? (2) Which ecological paradigms are most frequently employed and how are they conceptualized? (3) How are these ecological paradigms operationalized to investigate learning and developmental processes?

**Methods:**

A theory-informed narrative synthesis was used to analyze 70 studies published in Frontiers in Psychology. Studies were selected based on their explicit engagement with ecological perspectives, and were categorized according to educational context, target population, research topic, methodology, and ecological paradigm. The review focused on identifying patterns of theoretical use and operationalization within the studies, employing a narrative synthesis approach to integrate diverse studies and concepts.

**Results:**

The analysis revealed that most studies were conducted within higher education, with a predominant focus on student populations and topics such as motivation, mental health, and academic achievement. The studies identified three primary ecological paradigms: Bronfenbrenner's ecological systems theory, other ecological frameworks, and ecological validity as a methodological principle. A clear trend toward the increasing use of Bronfenbrenner's theory was observed. The studies leaned heavily on quantitative and multilevel techniques, with ecological paradigms primarily functioning as classificatory lenses. Findings reveal a shift from methodologically focused uses of ecological validity toward the consolidation of ecological systems theory, alongside emerging process-oriented and integrative approaches.

**Discussion:**

The review highlights the journal's contribution to advancing context-sensitive, relational, and temporally informed research, while also noting variation in the depth of theoretical engagement and operationalization. By situating these patterns within broader debates on ecological pluralism and theoretical clarification, the review offers conceptual and methodological insights for the continued refinement of ecological perspectives in educational psychology within increasingly complex educational environments.

## 1 Introduction

Educational psychology is concerned with understanding how individuals learn, develop, and adapt within educational settings, and how cognitive, motivational, social, and contextual factors jointly shape educational processes and outcomes (Alexander and Winne, 2012; Berliner, 2012). Over time, the field has expanded beyond a primary focus on individual learners to engage more explicitly with the contexts in which learning occurs, including classrooms, families, institutions, and broader sociocultural environments. This shift reflects growing recognition that educational phenomena cannot be adequately understood when abstracted from the conditions of everyday learning and development.

Ecological perspectives have been particularly influential in advancing this contextual turn. Originating in biology, *ecology* refers to the study of the dynamic and reciprocal relations between organisms and their environments (Haeckel, 1866; Heft, 2013). In psychology, ecological approaches conceptualize behavior, learning, and development as emerging from ongoing interactions between individuals and the material, social, and cultural features of their environments.

Within educational psychology, ecological perspectives encompass a range of theoretical and methodological orientations, including Bronfenbrenner's ecological and bioecological theories of human development (Bronfenbrenner, 1979; Bronfenbrenner and Morris, 2006), Gibson's ecological psychology and the concept of affordances (Gibson, 1979/2014), ecological validity as a guiding principle for designing research that reflects learners' everyday experiences (Brunswik, 1955), and community- and systems-oriented ecological frameworks concerned with interdependence, adaptation, and change (Kelly, 1986, 2006). Although these approaches differ in scale, emphasis, and application, they share a commitment to relational analysis, contextual embeddedness, and the study of learning and development *in situ*. Together, they offer powerful alternatives to decontextualized and purely intrapsychic models of educational phenomena.

Within this broader landscape, *Frontiers in Psychology* has played a distinctive role in advancing ecological thinking in educational psychology. Through its dedicated Educational Psychology section and interdisciplinary orientation, the journal has provided a platform for diverse ecological frameworks and perspectives, ranging from early work on ecological validity to extensive applications of Bronfenbrenner's Ecological Systems Theory (EST), as well as emerging integrative perspectives linking ecology with resilience, agency, wellbeing, and neuro-ecological critique. Collectively, these contributions reflect a growing and evolving constellation of ecological approaches that have shaped how educational phenomena are theorized and investigated.

Existing review studies have explored the use of Bronfenbrenner's bioecological theory in developmental science, particularly in family studies and child development (Tudge et al., 1996, 2016) and in international and intercultural education research (Tong and An, 2024). These reviews have documented persistent shortcomings in how the theory is applied across empirical studies, including the pervasive reliance on the earlier EST (Bronfenbrenner, 1979) at the expense of the more comprehensive bioecological model of Process-Person-Context-Time (PPCT) (Bronfenbrenner and Morris, 1998, 2006), as well as the partial or superficial representation of Bronfenbrenner's theory. However, these reviews were limited to Bronfenbrenner's framework and did not examine the broader range of ecological perspectives that have gained currency in educational research. The present study builds on and extends this critical tradition by asking whether similar patterns of conceptual inconsistency and selective application are evident within educational psychology more broadly, and whether other ecological frameworks are subject to comparable limitation.

This study is timely because the expansion of ecological perspectives has brought increased conceptual diversity and variation in application. Ecological perspectives differ in their ontological assumptions, analytical foci, and methodological implications, and their uptake reflects diverse disciplinary traditions and research priorities. This diversity signals both the breadth of ecological thinking and the need for systematic synthesis and conceptual clarification. Without such synthesis, there is a risk that ecological terminology may be applied inconsistently or conflated across theoretical and methodological traditions, obscuring how ecological perspectives contribute to understanding learning and development. The present review responds directly to this need, complementing earlier focused critiques by mapping the conceptual landscape of ecological scholarship as it has emerged in educational psychology over the past decade.

Accordingly, this review addresses the following research questions:

(1) What educational contexts, populations, research topics, and methodological approaches characterize this body of research?
(2) Which ecological paradigms are most frequently employed in educational psychology publications in *Frontiers in Psychology*?
(3) How are these ecological paradigms operationalized to investigate different dimensions of learning and development?

To address these questions, this review analyses 70 articles published between 2013 and 2025 that explicitly engage ecological frameworks in educational psychology. The review makes three main contributions: (1) it provides a comprehensive synthesis of ecological approaches as represented in *Frontiers in Psychology*; (2) by situating individual studies within broader ecological traditions, it clarifies the pluralistic nature of ecological thinking and offers conceptual guidance for selecting and combining ecological frameworks in future research; (3) by linking theoretical and methodological diversity with patterns of application, it identifies emerging paradigms and methodological opportunities for advancing ecological research in educational psychology.

The remainder of the article is organized as follows. Section 2 reviews the theoretical foundations and comparative perspectives of ecological approaches in educational psychology. Section 3 outlines the review methodology, including search procedures, inclusion criteria, and the coding framework. Section 4 presents findings on educational contexts, populations and methodological trends, while Section 5 reports findings on patterns of ecological framework use. Section 6 discusses the theoretical, methodological, and practical implications of these findings, and Section 7 concludes by highlighting future directions for ecological inquiry in educational psychology.

## 2 Literature review: ecological perspectives in educational psychology

### 2.1 The ecological turn in educational psychology

The ecological turn in educational psychology emerged as a sustained critique of the field's long-standing emphasis on decontextualised, individual-centered explanations of learning, development, and behavior. For much of the twentieth century, mainstream psychological research was characterized by what Bronfenbrenner famously described as the study of “the strange behavior of children in strange situations with strange adults” (Bronfenbrenner, 1977, p. 513). This tradition prioritized experimental control and intrapsychic processes while marginalizing sociocultural, historical, and environmental influences on development.

Ecological perspectives have their intellectual roots in biology and the study of organism–environment relations, most notably in the work of Haeckel in the late nineteenth century, but were gradually taken up across social sciences, including sociology, anthropology, and human geography (Tudge et al., 1996). Within psychology, early ecological sensibilities can be traced to pragmatist and holistic traditions, including the writings of Dewey, Lewin, and the Gestalt psychologists, all of whom emphasized the inseparability of individuals and their environments. As Heft (2013) argues, ecological psychology is best understood as an alternative to atomistic and intrapsychic models, grounded in a Darwinian emphasis on dynamic, mutually constitutive relations between organisms and their environments. From this perspective, ecological thinking rejects the mind–environment dichotomy inherited from associationist and Spencerian traditions and instead adopts a relational frame of analysis in which meaning, action, and development emerge through engagement with an econiche.

The ecological turn gained particular momentum in educational and school psychology as scholars increasingly questioned the explanatory and practical limitations of medical and deficit-oriented models (Gutkin, 2012; Williams and Greenleaf, 2012). Over time, this perspective informed prevention science, health promotion, and school-based interventions, positioning ecology as a foundational framework for understanding educational processes (Scarpa and Trickett, 2022).

### 2.2 Why ecological perspectives matter for educational psychology

Ecological perspectives offer significant conceptual, methodological and practical value for educational psychology by fundamentally reframing how learning, development, and behavior are understood.

Rather than locating educational difficulties within individuals, ecological approaches conceptualize learning as emerging from dynamic and reciprocal interactions between persons and their environments, thereby challenging deficit-oriented and medicalized models of explanation (Gutkin, 2012). Ecological perspectives also foreground the cultural, material, and institutional conditions that shape educational experience, providing conceptual resources for examining inequality, marginalization, and variation within schooling systems (Williams and Greenleaf, 2012).

Methodologically, ecological approaches encourage research designs that privilege contextual validity, longitudinal analysis, and naturalistic inquiry, moving beyond short-term, decontextualized experiments (Trickett and Rowe, 2012). In practice, this orientation supports a shift from remediation to prevention and from individual treatment to system-level intervention, positioning educational professionals as agents of ecological change (Williams and Greenleaf, 2012).

### 2.3 Major ecological traditions in educational psychology

Despite shared foundational commitments to contextualized and relational analysis, ecological approaches do not constitute a single, unified theory. As several scholars have noted, *ecology* in psychology functions as a broad conceptual orientation that has been variously interpreted and operationalized across subfields (Heft, 2013; Scarpa and Trickett, 2022).

Two theoretical lineages have been particularly influential: Bronfenbrenner's ecological and bioecological theories of development and Gibson's ecological psychology. In addition, community-based ecological models have contributed applied perspectives that emphasize contextual fit, interdependence, and institutional change.

Bronfenbrenner conceptualized development as embedded within a nested system of interacting contexts, ranging from immediate settings (microsystems) to broader sociocultural structures (macrosystems) (Bronfenbrenner, 1993). In his later work, he increasingly emphasized *proximal processes*, referring to enduring, reciprocal interactions between individuals and their environments, as the primary engines of development within the Process–Person–Context–Time (PPCT) model (Bronfenbrenner and Morris, 2006). This framework redirected attention from static contextual layers to the quality and meaning of interactions unfolding over time.

By contrast, Gibson's ecological psychology foregrounded perception and action, introducing the concept of affordances to describe the action possibilities that environments offer to individuals relative to their capabilities (Gibson, 1979/2014). From this perspective, meaning is not internally constructed but directly perceived through active engagement with the environment. Although originally developed in perceptual psychology, this approach has gained relevance for educational contexts, particularly in relation to learning environment design and embodied cognition.

Ecological perspectives in educational psychology have also been shaped by community psychology, which conceptualizes schools as complex social systems (Trickett and Rowe, 2012). It emphasizes multilevel analysis, system-level change, and the co-evolution of individuals and institutions, particularly in applied and intervention-oriented research (Scarpa and Trickett, 2022; Trickett and Rowe, 2012).

### 2.4 Comparative analyses of ecological theories

A growing body of scholarship has compared ecological theories to clarify both shared premises and substantive differences. Tudge et al. (1996) demonstrated that although both Gibson and Bronfenbrenner reject reductionist and intrapsychic explanations, they operate at different analytical levels and focus on distinct developmental mechanisms. Gibson's work addresses real-time perception and action, whereas Bronfenbrenner's framework is oriented toward long-term development within sociocultural systems. Comparative work has also examined intersections between Bronfenbrenner's theory and community psychology, showing that despite shared systems thinking, these frameworks diverge in their theoretical priorities and implications for practice (Scarpa and Trickett, 2022).

Recent comparative analyses have also highlighted internal diversity within ecological psychology. Bruineberg et al. (2024), for example, identify physical, biological, and social strands within ecological psychology, each grounded in different ontological and explanatory commitments. These distinctions are not intended to adjudicate between ecological theories, but to provide an analytic vocabulary for examining how different ecological paradigms are conceptualized and operationalized in educational psychology research.

### 2.5 Summary

This section demonstrates that ecological perspectives have played an increasingly visible role in educational psychology, offering relational and context-sensitive alternatives to individualistic and decontextualized models of learning and development. At the same time, ecology in educational psychology is not a single framework but a constellation of theoretical traditions that differ in scope, analytical focus, and methodological implications.

The conceptual diversity of ecological perspectives has important implications for reviewing ecological research. Without clear analytic distinctions, there is a risk of conflating conceptual reference with substantive empirical engagement. A theory-informed approach to synthesis is therefore necessary to examine not only whether ecological concepts are invoked, but how they are operationalized in research design, analysis, and interpretation. Recognizing this pluralism provides a crucial foundation for systematically examining how ecological approaches have been conceptualized and applied within *Frontiers in Psychology*, which is the focus of the analyses that follow.

## 3 Methods

### 3.1 Review design

This review adopted a theory-informed narrative synthesis with systematic elements to examine how ecological frameworks have been conceptualized and operationalized in educational psychology research published in *Frontiers in Psychology*. The primary aim was not to aggregate empirical effect sizes, but to analyze patterns of theoretical and methodological use, including how ecological paradigms have informed research design, measurement, and interpretation across diverse studies.

The reviewed literature exhibited substantial heterogeneity in theoretical orientations, research designs, analytical strategies, educational contexts, and target populations. In such circumstances, statistical synthesis is neither methodologically appropriate nor theoretically meaningful. Narrative synthesis has been widely recognized as a suitable approach for integrating heterogeneous bodies of evidence, particularly when the goal is conceptual clarification and explanatory insight rather than effect estimation (Popay et al., 2006; Snilstveit et al., 2012).

Importantly, the synthesis was explicitly theory-informed, using ecological frameworks as an analytic lens rather than treating them as background context. Theory-informed and interpretive approaches to evidence synthesis enable reviewers to move beyond descriptive summarisation and critically examine how theoretical assumptions are mobilized, adapted, or constrained across studies (Dixon-Woods et al., 2006). This approach aligns with the present review's focus on understanding the role of ecological paradigms in shaping contemporary educational psychology research.

### 3.2 Search strategy

To systematically identify publications employing ecological perspectives in educational psychology, a structured search was conducted within the *Frontiers in Psychology* journal database. The search focused exclusively on articles published in the Educational Psychology section of the journal. The search covered the entire publication period from the journal's inception to the end of 2025, which corresponds to the point at which data collection for this review was completed. Although the search window encompassed the full temporal range, eligible articles were published between 2013 and 2025.

Search terms were designed to capture studies that explicitly engaged with ecological frameworks or terminology, including *ecological, ecology*, and *ecosystem*. The search was conducted using the journal's internal search function and cross-checked manually to ensure coverage and consistency. This strategy was intentionally adopted to minimize researcher bias in selectively privileging particular educational theories as search keywords. However, the approach is limited by its reliance on terms containing the “eco-” prefix, meaning that ecologically informed research (e.g., sociocultural theory and situated learning) that does not explicitly employ such terminology may fall outside the scope of this review. This limitation is discussed in more detail in the Discussion section.

The first author and the third author independently screened the articles against the inclusion and exclusion criteria; their decisions were then compared, yielding an agreement rate of 90%. Discrepancies were resolved through consensus discussion among all three authors.

### 3.3 Inclusion and exclusion criteria

Given the review's aim to map how ecological perspectives have been explicitly conceptualized and mobilized within *Frontiers in Psychology*, inclusion and exclusion decisions were guided primarily by paradigmatic orientation and publication type, rather than by journal article labels alone.

Articles were included if they met both of the following criteria:

- (1) Paradigmatic orientation: The article explicitly adopted, referenced, or discussed an ecological perspective, framework, or concept, as indicated by the use of the keywords *ecological, ecology*, or *ecosystem* in the title, abstract, keywords, or conceptual framing.
- (2) Publication type: The article was published as an empirical study or a theoretical paper. Theoretical papers were included when they advanced an ecological framework or used it as a primary analytic lens, rather than merely mentioning ecological concepts in passing.

Articles were excluded if they met any of the following criteria:

(1) Superficial use of ecological terminology: Studies in which the terms *ecological, ecology*, or *ecosystem* appeared only incidentally (e.g., in background, discussion, or future directions) without informing the study's conceptual framing, research design, or analytical approach.
(2) Review articles, meta-analyses and commentaries: Review articles and meta-analyses (e.g., Kenny et al., 2023; Li et al., 2022; Tong and An, 2024) and articles explicitly categorized as commentaries (e.g., Hu and Mei, 2022) were excluded, as the focus of this review was on the use of ecological frameworks in original empirical or theoretical studies.

### 3.4 Screening and final corpus

All retrieved articles were screened in two stages. First, titles and abstracts were examined to confirm explicit engagement with ecological terminology and publication type. Second, full texts were reviewed to verify paradigmatic positioning and alignment with the inclusion criteria. Through this process, a final corpus of 70 articles was identified for analysis.

### 3.5 Analytical approach: theory-informed narrative synthesis

The analysis proceeded through three interconnected phases, each designed to address the review's research questions while remaining sensitive to the theoretical and methodological diversity of ecological approaches. This phased approach aligns with established guidance on conducting narrative synthesis in systematic reviews (Popay et al., 2006; Snilstveit et al., 2012).

#### 3.5.1 Phase 1: descriptive mapping

The first phase involved systematic extraction and coding of study characteristics to establish an overview of the empirical, topical, and methodological landscape of the corpus. Specifically, this phase examined publication year, educational contexts, populations, research topics, and methodological approaches, providing the empirical foundation for addressing RQ1. Such descriptive mapping is recognized as a foundational step in narrative synthesis, enabling reviewers contextualize subsequent interpretive analysis (Petticrew and Roberts, 2006).

#### 3.5.2 Phase 2: conceptual and paradigmatic classification

The second phase focused on identifying and classifying how ecological perspectives were conceptualized across the included studies. Specifically, studies were classified into three broad categories inductively: (a) Bronfenbrenner's ecological or bioecological theory; (b) other ecological theories or frameworks (e.g., ecological psychology, social-ecological models, agency ecology); and (c) ecological validity conceptualized as a methodological principle rather than as a substantive theory. Where applicable, further distinctions were made within theory-based categories, such as between classic ecological systems theory and the bioecological PPCT model.

Notably, inclusion of ecological validity alongside theory-oriented ecological perspectives reflects its recurrent use in the reviewed studies as a distinct way of operationalizing “ecological” thinking in educational research. In this sense, ecological validity is analyzed as a methodological orientation within the broader landscape of ecological perspectives, allowing for a more comprehensive account of how ecological concepts are taken up in practice.

This phase directly addressed RQ2 concerning the ecological paradigms most frequently employed in educational psychology research published in *Frontiers in Psychology* and how these paradigms are conceptualized.

#### 3.5.3 Phase 3: pattern-based synthesis

The third phase moved beyond paradigmatic classification to synthesize how ecological perspectives were operationalized to investigate learning and development. This phase analyzed patterns in ecological systems/levels/processes examined and the function that ecological perspectives served within studies (e.g., as a classificatory lens or as a methodological principle).

Patterns were identified through iterative reading and constant comparison across studies, guided by the conceptual distinctions established in the literature review. This analytically informed approach follows recommendations for theory-informed synthesis that treats theoretical frameworks as analytical tools rather than solely as descriptive labels (Barnett-Page and Thomas, 2009; Dixon-Woods et al., 2006). This phase directly addressed RQ3, which asks how ecological paradigms are operationalized to investigate different dimensions of learning and development.

Throughout the analysis, attention was paid to both convergent patterns and notable variations in ecological framework use. Rather than imposing a single evaluative standard, the synthesis adopted a pluralist stance that recognized the legitimate diversity of ecological approaches while also identifying recurring patterns, constraints, and areas for further theoretical development (Barnett-Page and Thomas, 2009).

### 3.6 Coding scheme

To support the analytical approach described above, a structured coding scheme was developed to ensure systematic and transparent data extraction across the included studies (Hong et al., 2017). The scheme comprised three domains: (a) study characteristics, (b) paradigmatic orientation, and (c) operationalization patterns.

#### 3.6.1 Coding of study characteristic

The study characteristics domain supported Phase 1 of the analysis. Studies were coded across six dimensions: educational setting, target population, geographic location, research topic, research methodology and analytical method. These dimensions were selected based on standard reporting guidelines for educational research (American Educational Research Association, 2006).

#### 3.6.2 Coding of ecological orientation

The paradigmatic orientation domain supported Phase 2 and was structured hierarchically.

At Level 1, studies were coded by ecological paradigm type. Drawing on the comparative literature reviewed in Section 2 and patterns identified in the corpus, studies were categorized according to their primary paradigmatic orientation, distinguishing among:

(1) Bronfenbrenner-based theory,
(2) other ecological frameworks, and
(3) ecological validity conceptualized as a methodological principle.

For studies coded as “other ecological frameworks,” the specific ecological tradition was identified where explicitly stated (e.g., Gibson's ecological psychology, social-ecological models, agency ecology, ecological momentary assessment approaches).

#### 3.6.3 Coding of operationalization patterns

The operationalization patterns domain supported Phase 3 by capturing how ecological paradigms were enacted analytically. This domain included:

(1) versions of Bronfenbrenner-based theory (classic ecological systems theory [EST], bioecological PPCT model, or integrated/partial use), following established periodization's of Bronfenbrenner's theoretical development (Tong and An, 2024; Tudge et al., 1996, 2016).
(2) system levels examined (micro-, meso-, exo-, macro-, and chronosystem components),
(3) analytic functions, that is, how the ecological paradigms operated within the study's analytic logic (e.g., classificatory lens, explanatory framework for processes, methodological principle, or platform for integration).

The identification of analytic functions emerged inductively from preliminary analysis, consistent with framework synthesis approaches that allow categories to develop through engagement with the data (Carroll et al., 2013).

To ensure analytic consistency, system levels were coded conservatively. A system level was considered examined only when it was operationalized through explicit variables, data sources, or analytic claims directly linked to that level. In some studies, system levels were conceptually implied or invoked in the interpretation of findings without being directly operationalized. These instances were distinguished from analytically examined levels and were coded as referenced only. This distinction was intended to differentiate between rhetorical acknowledgment of ecological systems and their substantive empirical engagement. This conservative coding strategy aligns with prior critiques of ecological research that caution against conflating conceptual reference with analytic use (Bronfenbrenner and Morris, 2006; Tudge et al., 1996).

Coding was initially conducted by a trained research assistant using a standardized protocol. To ensure reliability, a random sample of 14 studies (19.7%) was independently coded by one of the researchers, with inter-rater agreement exceeding 0.80 (Cohen's kappa) for all categorical variables, indicating substantial agreement (Landis and Koch, 1977). All three researchers reviewed the final coding, and discrepancies were resolved through discussion until consensus was reached.

## 4 Findings I: descriptive mapping—educational contexts, populations, topics, and methodological trends

This section addresses RQ1 by examining the descriptive data that characterize ecological research in *Frontiers in Psychology*. Beyond documenting the distribution of these research characteristics, we identify patterns and gaps that warrant attention in future inquiry.

### 4.1 Educational contexts

The reviewed studies were situated across a range of educational contexts, though the distribution was notably uneven (see Table 1).

**Table 1:** Distribution of educational settings (*N* = 70).

Setting	*n*	%
Higher education	23	32.9%
Secondary school (mixed)	10	14.3%
Senior high school	5	7.1%
Junior high school	4	5.7%
Primary school	2	2.9%
Primary and secondary schools	3	4.3%
Preschool/Early childhood	10	14.3%
Children (mixed ages)	7	10%
Other/unspecified	6	8.6%

Higher education settings were clearly dominant, accounting for 23 studies (32.9%, combining student-focused and teacher-focused research). These studies examined diverse topics, including students' resilience (Sheng et al., 2025), academic engagement (Li et al., 2025; Liang and Wu, 2025), international students' experiences (Zhang et al., 2023), and teachers' professional development (Jiang et al., 2022; Ren and Zhou, 2023).

Apart from higher education, secondary education contexts constituted another substantial portion of the corpus. Studies in secondary education, including the lower/junior and upper/senior segments, addressed topics such as adolescents' mathematics achievement in relation to parenting styles (Wang et al., 2024) and school belonging and reading literacy (Tan et al., 2022). Studies in early childhood (preschool) and primary education settings focused on children's social behavior problems (e.g., Liu et al., 2024; Wang et al., 2023), early childhood curiosity (Shah et al., 2023), vocabulary development (Southwood et al., 2021), shared storybook reading (Grolig, 2020) and students' physical fitness (Li and Cheong, 2022).

A small subset of studies (6 studies, 8.6%) was classified as *Other/Unspecified*. This category captures research conducted outside formal schooling contexts or across non-traditional educational ecologies, including laboratory-based studies not situated within institutional educational settings (e.g., Kalbfleisch et al., 2013), studies situated in transnational cultural-educational contexts such as Confucius Institutes (e.g., Wang I. K. -H., 2022), and work examining learning and development in non-educational professional settings such as the business/entrepreneurship context (e.g., Sargani et al., 2021). The presence of these studies highlights the conceptual breadth of ecological approaches, which extend beyond formal schooling to encompass learning and development across diverse institutional and sociocultural environments.

Despite this breadth, several educational contexts remained underrepresented. Vocational education appeared in only one study (i.e., Liu et al., 2022), while informal learning, community-based education, and workplace learning were largely absent from the corpus. This concentration on formal educational institutions suggests potential gaps in the coverage of everyday and non-institutional learning contexts.

### 4.2 Target populations

The populations examined in ecological research exhibited both expected patterns and notable gaps.

#### 4.2.1 Students as primary participants

Across all educational levels, students constituted the most frequently studied population, appearing in 49 studies (70%) (see Table 2). These studies encompassed learners across the educational lifespan, including pre-school children, primary and secondary school students, and university students.

**Table 2:** Distribution of targeted populations (*N* = 70).

Population	*n*	%
Students	49	70.0%
Teachers	16	22.9%
Other	5	7.1%

A distinctive feature of the corpus, however, was its substantial attention to teachers, who appeared as primary participants in 16 studies (22.9%). Teacher-centric studies examined in-service teachers' emotions across different career stages (e.g., Han et al., 2023; Lai et al., 2024; Lei et al., 2022; Shi et al., 2023; Yao et al., 2023), professional agency in curriculum reform contexts (e.g., Gao and Cui, 2022; Jiang et al., 2022; Wang L., 2022), and stress among teachers of gifted students (e.g., Lv et al., 2024). Collectively, these studies highlight the journal's openness to ecological analyses of teacher development and wellbeing as situated within multilevel educational systems.

A limited number of studies attended to populations facing specific challenges: migrant children (e.g., Ding et al., 2024; Xu and Lv, 2022), international students (e.g., Zhang et al., 2023), and children with disabilities (e.g., Mahmic et al., 2021; Nicholas, 2020). Although limited in number, such studies demonstrate the potential of ecological frameworks for examining experiences shaped by mobility, marginalization, and different access to resources.

The *Other* category (5 studies, 7.1%) comprised research that did not center on a single, clearly defined learner group. This included studies involving multiple academic stakeholder groups within broader academic ecologies (e.g., Burr et al., 2022), research situated in entrepreneurial ecosystems focusing on technology entrepreneurs (e.g., Sargani et al., 2021), and theoretical or conceptual papers in which the target population was intentionally general or unspecified (e.g., Nguyen and Doan, 2025). This category underscores the ecological paradigm's capacity to extend beyond conventional learner populations and formal educational roles.

#### 4.2.2 Geographic concentration

A striking feature of the reviewed literature was the geographic concentration of research participants (see Table 3).

**Table 3:** Geographic distribution of studies (*N* = 70).

Region	*n*	%
China	49	70.0%
Europe	11	15.7%
USA	4	5.7%
South Africa	1	1.4%
Australia	1	1.4%
**Unspecified**	4	7.1%

Studies conducted in China constituted approximately 70% of the corpus, indicating a pronounced geographic concentration. While this reflects China's growing research productivity in educational psychology and demonstrates the applicability of ecological frameworks in Chinese contexts, this pattern suggests potential limitations in the cross-cultural generalizability of the findings.

Europe represented the second most frequent research context (15.7%), though this category included heterogeneous cases. Nine studies were conducted within a single European country (4 in Spain and 1 each in Italy, Ireland, Portugal, Germany, Czech, and the UK), while one study drew on data from 16 European countries as part of a large-scale, cross-national project (Milosevic et al., 2022).

Finally, 7.1% of the reviewed studies did not specify a national context. These papers were predominantly theoretical or conceptual in nature (e.g., Nguyen and Doan, 2025; Nicholas, 2020), or addressed populations in general terms rather than situating analysis within a specific country (e.g., Yuan and Wang, 2022).

### 4.3 Research topics

Analysis of research topics revealed the substantive foci of ecological research in educational psychology (see Table 4). Topics were coded as non-mutually exclusive, allowing studies to address multiple themes.

**Table 4:** Distribution of research topics (*N* = 70, multiple selection).

Topic	*n*	%
Motivation and engagement	61	87.1%
Mental health/emotion	43	61.4%
Technology and media	40	57.1%
Adaptation and adjustment	35	50.0%
Career development	30	42.9%
Academic achievement	24	34.3%
Language learning	16	22.9%
Resilience	10	14.3%
Teacher development	8	11.4%
Social development	8	11.4%
Creativity	7	10.0%
Special education	6	8.6%
Home-school collaboration	5	7.1%

Motivation and engagement emerged as the most prevalent research focus, appearing in 61 studies (87.1%), followed by mental health and emotional outcomes (43 studies, 60.6%) and technology-mediated learning (40 studies, 57.7%). This topical distribution is consistent with contemporary concerns in educational psychology, particularly the growing emphasis on learner wellbeing and digital learning environments. The relatively lower attention to home-school collaboration (7.1%) is notable given that mesosystem interactions represent a distinctive contribution of the ecological perspective.

### 4.4 Methodological orientations

Table 5 presents the distribution of research methodologies, showing that quantitative methods predominated, appearing in 41 studies (58.6%), followed by mixed-methods designs (17 studies, 24.3%) and qualitative approaches (9 studies, 12.9%). Only a small number of theoretical or conceptual studies (3 studies, 4.3%) did not clearly specify an empirical methodological orientation.

**Table 5:** Distribution of research methodologies (*N* = 70).

Methodology	*n*	%
Quantitative	41	58.6%
Mixed methods	17	24.3%
Qualitative	9	12.9%
Unspecified	3	4.3%

The analytical methods employed across the corpus reflected sophisticated engagement with multilevel data structures. Structural equation modeling (SEM) appeared in 26 studies (36.6%, including those combining SEM with multilevel modeling), multilevel modeling in 14 studies (19.7%), and regression-based analysis in nine studies (12.7%).

### 4.5 Summary

The analysis reveals patterns of consolidation as well as areas of potential development in ecological research in educational psychology. The strong presence of studies in higher education and secondary school contexts, along with the prominence of student-focused research, indicates a strong focus on key educational populations and settings. At the same time, the relative absence of work in vocational, informal, and cross-cultural contexts points to promising directions for extending ecological inquiry. Methodologically, the widespread use of quantitative and multilevel techniques reflects increasing analytical sophistication, while also signaling opportunities to more fully align research designs with the ecological perspective by incorporating longitudinal, mixed-methods, and process-oriented approaches. Overall, these patterns indicate scope for further research to engage more extensively with dynamic, multi-system, and temporally oriented dimensions of learning.

## 5 Findings II: ecological paradigms represented in the reviewed studies

In responding to RQ2, the reviewed literature revealed three major categories of ecological paradigms: Bronfenbrenner's ecological/bioecological theory (41 studies, 58.6%), other ecological frameworks (19 studies, 27.1%), and ecological validity as a methodological principle (10 studies, 14.3%). The 19 studies employing ecological frameworks beyond Bronfenbrenner included Gibson's ecological psychology/affordances (6 studies), social-ecological perspectives on teacher agency (2 studies), ecological momentary assessment (1 study), and various domain-specific frameworks such as media ecology, reading ecosystems, and neuro-ecological approaches. This diversity reflects both the dominance of Bronfenbrenner's theory and the breadth of ecological thinking in contemporary educational psychology research.

**Figure 1:**
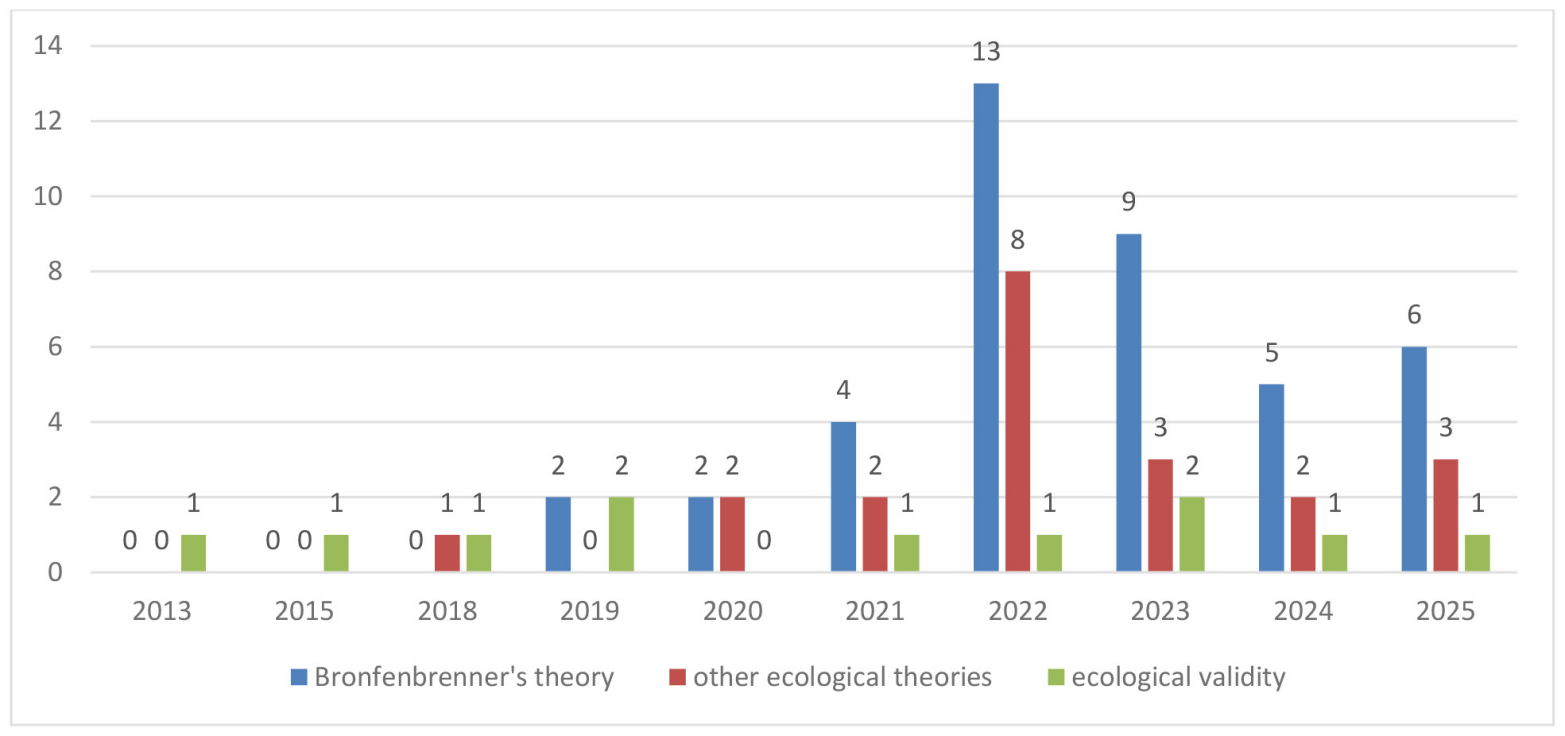
Temporal distribution of different ecological paradigms.

A further examination of publication trends over time (Figure 1) reveals a clear shift in how ecological perspectives have been conceptualized. In the early years of the review period (2013–2017), contributions were sparse and primarily methodologically oriented, with isolated studies invoking ecological validity appearing in 2013 and 2015, and no explicit engagement with ecological theory during this initial period. From 2018 onward, ecological perspectives began to diversify modestly, marked by the first appearance of other ecological theories alongside continued attention to ecological validity. A more pronounced expansion occurred from 2019 to 2021, during which publications drawing on Bronfenbrenner's ecological systems theory began to increase steadily and were accompanied by a growing, though smaller, body of work engaging alternative ecological frameworks. The most substantial growth is evident in 2022, which represents a peak year across all ecological paradigms, particularly for Bronfenbrenner-based studies, indicating a period of consolidation and intensified theoretical engagement. Although publication numbers declined slightly after 2022, ecological research remained consistently visible through 2025, with Bronfenbrenner's theory continuing to dominate and other ecological approaches maintaining a stable presence.

These trends suggest an evolution from predominantly methodological uses of ecological validity toward a more theory-driven and diversified ecological landscape within *Frontiers in Psychology*, indicating increased visibility of ecological perspectives within the journal over time. Taken together, these findings indicate that ecological paradigms in *Frontiers in Psychology* are characterized by a clear paradigmatic asymmetry. Bronfenbrenner's ecological systems theory functions as the dominant organizing framework, while other ecological approaches appear more sporadically and are often adapted to domain-specific research questions. Ecological validity, in contrast, is consistently employed as a methodological orientation rather than as a substantive theoretical lens.

## 6 Findings III: patterns of ecological perspectives and analytic operationalisation

RQ3 examines how ecological paradigms were operationalized. Within the categories identified above, ecological perspectives functioned as: (1) classificatory lenses for organizing multilevel predictors of educational outcomes; (2) conceptual frameworks for examining developmental processes; (3) methodological principles emphasizing ecological validity; and (4) platforms for theoretical integration with other psychological and educational theories. The following subsections synthesize these recurring patterns of use.

### 6.1 As classificatory lenses for multilevel predictors

The reviewed literature reveals a marked tendency to operationalize Bronfenbrenner's classic EST primarily as a classificatory framework. Among the 41 studies drawing on Bronfenbrenner's theory, 24 (58.5%) relied primarily on the EST model, 16 (39.0%) selectively integrated elements of the PPCT framework, and only one study (2.4%) employed the bioecological model as its central analytic framework (i.e., Grolig, 2020). Across these studies, EST functioned mainly to organize variables across nested environmental levels and to justify their inclusion in statistical models.

Analysis of system levels further revealed the dominant system-level components investigated. Twenty studies (48.8%) examined multiple system levels (three or more), with 10 studies (24.4%) addressing all five levels including the chronosystem. Thirteen studies (34.1%) focused exclusively on microsystem factors, and two studies (4.9%) did not clearly specify which system levels were examined, as ecological systems functioned primarily as rhetorical devices rather than as analytically distinct system levels (Dou et al., 2022; Hong et al., 2022).

#### 6.1.1 Dominance of microsystem components

Across this body of research, microsystem-level predictors dominated empirical models. Among all Bronfenbrenner-informed studies, 37 (90.2%) examined microsystem-level factors. Analysis of microsystem components revealed that school/teacher factors appeared in all 41 studies (100%), family factors in 37 studies (90.2%), peer factors in 25 studies (61.0%), and community factors in 26 studies (63.4%).

Family-related variables, including parental rearing styles, autonomy support, emotional support, were consistently positioned as proximal influences on learners' outcomes. For instance, Wang et al. (2024) investigated how parents' rearing styles predicted adolescents' mathematics achievement through the mediating roles of self-control and math anxiety. Likewise, Yang et al. (2025) examined how parental educational expectations influenced primary school students' academic achievement through the mediating role of parent-child communication and self-efficacy.

School-based interpersonal factors constituted another prominent microsystem focus. Teacher support, teacher-student relationships, and classroom climate were frequently examined as predictors of student outcomes (e.g., Foody et al., 2019; Huang et al., 2023; Liu et al., 2022). These variables often operated through mediating psychological processes such as motivation, self-control, or school belonging.

Peer-related factors were included in approximately 61% of microsystem-focused studies and, when examined, yielded more mixed associations with educational outcomes. For instance, Li et al. (2025) found that while teacher autonomy support was significantly positively associated with academic engagement, peer support showed a significant negative association. This finding complicates simplistic assumptions about microsystem influences. Huang et al. (2023) similarly reported that peer support had no significant association with adolescents' psychological and somatic health, while teacher support showed stronger effects.

#### 6.1.2 Emerging attention to mesosystem, exosystem, and macrosystem

Mesosystem influences, defined by Bronfenbrenner as interactions among microsystems (Bronfenbrenner, 1993), were addressed in a more limited number of recent studies and were often operationalized through composite or relational indicators. For example, Liu et al. (2024) investigated parent-teacher relationships and their associations with preschool children's social behavior problems, finding that work-family conflict and parenting self-efficacy played chain mediating roles. Similarly, Wang et al. (2023) examined how work-to-family conflict leads to preschool children's social behavior problems through the mediating roles of parenting guilt and parent-child relationships. These mesosystem variables were typically examined as indirect predictors, exerting influence through mediating factors.

Exosystem and macrosystem factors, such as neighborhood quality, institutional arrangements, educational policies, or sociocultural norms, were incorporated less frequently and often played a contextual or background role. For instance, neighborhood environment and regional context were occasionally included as exosystem or macrosystem indicators in large-scale or secondary-data analyses (e.g., Huang et al., 2023; Liu et al., 2021). In another example, Wei and Zhang (2025) explored factors affecting Australian students' mathematics self-efficacy, providing cross-cultural comparison of macrosystem influences. Broader cultural or policy contexts were more commonly discussed interpretively rather than empirically modeled.

Overall, this pattern indicates a pragmatic use of ecological frameworks that prioritizes conceptual coverage and analytical clarity. While such approaches support the development of context-sensitive models of educational outcomes, ecological levels were generally treated as relatively static categories, and cross-level relations were most often examined through mediation or moderation models rather than longitudinal or process-oriented designs. This pattern suggests that the dynamic and developmental dimensions of ecological systems theory remain less frequently operationalized.

### 6.2 As explanatory frameworks for developmental processes

A smaller but theoretically significant subset of studies employed ecological frameworks to examine processes such as emotions, agency, and resilience as unfolding through ongoing interactions between individuals and their environments. Rather than mapping static contextual predictors, these studies focused on how psychological phenomena emerge, evolve, and are sustained across time and ecological contexts. This pattern was most evident in research on teacher development and wellbeing, particularly in studies tracing emotional trajectories, professional agency, and adaptive responses to institutional and sociocultural change.

#### 6.2.1 Developments of teacher emotions

Several studies adopted ecological frameworks to investigate how teachers' emotional experiences emerge from their interactions with multiple environmental systems. For example, Lai et al. (2024) constructed a model of teachers' emotion generation drawing on emotional geography theory and Bronfenbrenner's ecological theory, identifying factors at personal, contextual, and sociocultural levels. Similarly, Sun and Yang (2021) employed a narrative case study approach to trace the emotional trajectories of two Chinese senior high school English teachers, revealing how emotions evolved through interactions with ecological systems during a teaching improvement program. Shi et al. (2023) conducted a case study of college English teachers' emotional labor, demonstrating how institutional contexts shape affective experiences.

#### 6.2.2 Trajectories of professional agency and identity

Teacher agency emerged as a significant theme within this pattern. Wang I. K. -H. (2022) examined English language teacher agency in response to curriculum reform, demonstrating how teachers navigate and negotiate ecological constraints. Jiang et al. (2022) adopted an ecological approach to understanding university EFL teachers' professional development, identifying how institutional, disciplinary, and policy contexts shape career trajectories. Other studies explored teacher identity formation (Wang et al., 2021) and factors contributing to research demotivation among college teachers (Ren and Zhou, 2023).

#### 6.2.3 Experiences of resilience

The COVID-19 pandemic catalyzed a surge of ecologically-oriented resilience research. For instance, Gao et al. (2022) investigated how language teachers' resilience co-evolved through intrapersonal and interpersonal reflections during the pandemic. Liaqat et al. (2025) adapted Bronfenbrenner's framework to examine academic resilience among undergraduate English learners in Pakistan, uncovering significant differences across ethnic groups. Zhang et al. (2023) documented international students' experiences during COVID-19 in U.S. higher education, revealing how the pandemic disrupted ecological support systems.

#### 6.2.4 The chronosystem in focus and methodological diversity

Unlike the first pattern, studies on emotions, agency, and resilience made substantial use of the chronosystem, which attends to how psychological phenomena unfold over time. Relatedly, this pattern exhibited greater methodological pluralism than the first. While quantitative approaches remained common, qualitative methods were employed in eight studies (11.3%) and mixed methods (e.g., Gao et al., 2022; Han et al., 2023) in 16 studies (22.5%), with case study designs appearing (e.g., Sun and Yang, 2021; Yao et al., 2023) appearing in 10 studies (14.1%) and longitudinal designs (e.g., Gao and Cui, 2022; Lei et al., 2022) in 15 studies (21.1%).

As can be seen, since studies in this cluster more frequently incorporated the chronosystem, they operationalize time through longitudinal designs and extended narrative accounts. For instance, Lei et al. (2022) tracked the emotions of two mathematics teachers over 4 years in a teaching improvement program, documenting phases of “mixed sadness and happiness” eventually transitioning to “delight and calmness” (p. 1). Gao and Cui (2022) followed a teacher leader's emotional trajectory over five years during an English for Academic Purposes reform, revealing how emotion, agency, power, and identity functioned as dynamically interrelated constructs. Likewise, Yao et al. (2023) examined two novice EFL teachers' emotional experiences over 3 years in rural primary schools, capturing how ecological contexts shape early career development.

This line of research contributed to ecological accounts in educational settings by foregrounding temporal dynamics and extended developmental trajectories. This methodological diversity is consistent with the ecological perspective's emphasis on situated, dynamic processes that unfold over time and across contexts. However, the processes through which ecological factors became developmentally consequential remained largely descriptive. In particular, Bronfenbrenner's notion of proximal processes (Bronfenbrenner and Morris, 1998), intended to explicate how development is produced through sustained, reciprocal interaction, was seldom explicitly operationalized.

### 6.3 As methodological principles emphasizing ecological validity

A third pattern involved the invocation of ecological validity as a methodological principle for research design and measurement rather than a substantive ecological theory, as evident in 10 studies (14.1%). Unlike the preceding patterns, which operationalized ecological theories to explain developmental processes, studies in this category employed the term “ecological” primarily to signify “real-world relevance” or “contextual authenticity” rather than an explicit theoretical commitment to understanding development within environmental systems.

#### 6.3.1 Design-level ecological validity

Several studies emphasized the importance of designing research tasks that authentically reflect learners' real-world environments. For instance, Rhodes et al. (2024) offered a conceptual framework for ecologically valid measurement of problem solving, arguing that without situating problem solvers, problem contexts, and researchers' own experiential partialities, research risks conflating information relevance with confirmatory biases. Matthes and Stoeger (2021) investigated whether implicit theories about ability predicted learning strategy use when assessed through behavior-proximal measures in ecologically valid classroom settings, contrasting this with typical self-report approaches.

#### 6.3.2 Technology-enhanced ecological validity

A subset of studies focused on the ecological validity of technology-based assessment tools. Fernandez et al. (2023) developed normative data for a virtual reality tool designed to evaluate executive functions in children and adolescents in a more ecological way. The authors argued that virtual reality environments can simulate real-world demands while maintaining experimental control. Chung et al. (2022) conducted an ecological validation study of a self-managed online mindfulness program implemented through a university's learning management system.

This line of research represents the continuation and refinement of methodologically oriented uses of ecological validity within educational psychology in the early years of the review period, particularly in the design of assessments. It contributes to strengthening the methodological rigor and contextual grounding of educational research while being limited by remaining analytically detached from broader ecological theories for explaining developmental processes and system-level interactions.

### 6.4 As platforms for theoretical integration

The fourth pattern is that ecological frameworks are often used in combination with other theories (e.g., social cognition, sociocultural theory, academic resilience) to explain complex psychological phenomena in education. The most frequent integration paired ecological systems theory with self-determination theory (SDT). For instance, Li et al. (2025) explicitly grounded their study in both frameworks, examining how autonomy support from different ecological sources (parents, teachers, peers) influenced academic engagement through the SDT-derived mechanisms of basic psychological needs and autonomous motivation. This integration is conceptually coherent: ecological theory specifies the environmental sources of support, while SDT specifies the psychological mechanisms through which support influences outcomes.

While these theoretical combinations represent valuable scholarly efforts, most instances involved juxtaposition rather than genuine integration. Researchers typically invoked multiple theories to justify variable selection or to interpret findings post hoc, but the theoretical tensions, complementarities, and potential syntheses between frameworks were rarely examined in depth.

### 6.5 Summary

This section has identified four prominent functional patterns of ecological frameworks. These patterns are not mutually exclusive; individual studies sometimes exhibited characteristics of multiple patterns. Nevertheless, the typology illuminates distinct ways in which “ecological” thinking has been operationalized in educational psychology research, along with the strengths and limitations associated with each approach.

## 7 Discussions and future directions

This review examined how ecological frameworks have been conceptualized and operationalized in educational psychology research published in *Frontiers in Psychology* between 2013 and 2025. By synthesizing 70 studies through a theory-informed narrative approach, the review highlights the journal's contribution to advancing ecological thinking in educational psychology, while also identifying productive directions for further theoretical and methodological refinement. In this section, we discuss how the reviewed work collectively illustrates the value of ecological perspectives, how different ecological traditions have been taken up in practice, and how future research might deepen alignment between ecological paradigms and empirical inquiry.

### 7.1 Consolidating ecological perspectives in educational psychology

A central contribution of the reviewed literature lies in the consolidation of ecological perspectives as a legitimate and increasingly influential orientation within educational psychology. As discussed in Section 2, ecological approaches matter precisely because they shift analytic attention from isolated individuals to relational, contextualized, and developmentally situated processes (Williams and Greenleaf, 2012). The corpus published in *Frontiers in Psychology* demonstrates how this shift has been translated into empirical research across a wide range of educational topics and settings.

Several contextual and disciplinary factors can be considered as possible explanations for the directionality and variability observed across the reviewed studies. The prominence of higher education research likely reflects both pragmatic considerations, such as the accessibility of university participants, and the growing scholarly interest in young adults' psychosocial adjustment during critical developmental transitions. The strong student-centric focus aligns with educational psychology's traditional emphasis on learner development, achievement, and psychosocial adjustment. The geographic concentration of studies conducted in China may similarly reflect both the country's growing research productivity in educational psychology and the cultural resonance of ecological theory's emphasis on family, school, and community within collectivist educational traditions. In addition, the dominance of cross-sectional, quantitative approaches aligns with longstanding disciplinary training traditions in educational psychology, which have historically emphasized variable-centered, statistically-driven analyses (Tudge et al., 2016).

In particular, studies drawing on Bronfenbrenner's ecological systems theory have enabled more context-sensitive modeling of educational phenomena. By organizing predictors across multiple system levels, these studies move beyond single-context explanations and foreground the embeddedness of learning and development within families, schools, and broader sociocultural environments (e.g., Li et al., 2025; Tan et al., 2022; Wang et al., 2024). Ecological systems theory represents a pragmatic advance over decontextualized approaches and reflects the field's growing recognition of ecological complexity.

However, ecological frameworks have most often been mobilized in classificatory rather than process-oriented ways. The widespread reliance on classic ecological systems theory, rather than the later bioecological PPCT model, likely reflects the former's conceptual simplicity and ease of operationalization as a variable-organizing framework. While this pattern has facilitated broader uptake of ecological perspectives, it also resonates with longstanding critiques that ecological theory is frequently reduced to a taxonomy of contexts rather than mobilized as a process-oriented theory of development (see Tong and An, 2024; Tudge et al., 1996). Addressing this limitation will require more explicit engagement with the processual core of Bronfenbrenner's bioecological mode, particularly the concept of proximal processes as engines of development.

Future work could build on these classificatory foundations by more explicitly engaging with interactional and developmental mechanisms. Greater engagement with the updated version of Bronfenbrenner's bioecological model is needed to move beyond static classification toward mechanistic explanation (Tong and An, 2024).

### 7.2 Strengths and extensions beyond the microsystem

Another notable contribution of the reviewed research is the accumulation of detailed empirical knowledge at the microsystem level. The overwhelming focus on family, teacher, classroom, and peer contexts has generated robust evidence on how proximal educational environments shape students' and teachers' psychological outcomes (e.g., Foody et al., 2019; Huang et al., 2023; Wang et al., 2024). This body of work exemplifies the practical relevance of ecological perspectives for educational psychology and provides a strong empirical foundation for intervention and policy. The predominance of microsystem-level analysis reflects the relative accessibility of proximal variables through established survey instruments, whereas mesosystem and macrosystem influences often require more complex, resource-intensive designs, such as longitudinal, multi-informant, or qualitative designs (e.g., Lei et al., 2022; Wang et al., 2025; Yao et al., 2023).

Beyond these strengths, the reviewed corpus also points to promising avenues for extending ecological analysis beyond the microsystem. Although mesosystem, exosystem, and macrosystem factors were less frequently operationalized, existing studies examining parent–teacher relationships, work–family dynamics, migration contexts, and cross-national differences demonstrate the feasibility of such analyses (e.g., Liu et al., 2024; Wei and Zhang, 2025; Xu and Lv, 2022).

Future research could build on these efforts by moving from treating higher-level contexts as background conditions toward analyzing them as dynamic and interacting systems. Such work would more fully capitalize on ecological theory's distinctive contributions: explaining how relations among systems shape educational development over time.

### 7.3 Advancing process-oriented and temporal ecological research

From a bioecological perspective, ecological analysis is expected to move beyond mapping contexts toward explicating the reciprocal processes through which development unfolds across time and systems (Bronfenbrenner and Morris, 2006). One of the most distinctive strengths of the journal lies in its support for research that engages ecological theory in a more process-oriented and temporally sensitive manner. Studies focusing on teacher emotions, agency, and resilience have employed qualitative, longitudinal, and mixed-methods designs to trace psychological development across time, institutional change, and crisis conditions (e.g., Gao and Cui, 2022; Lei et al., 2022; Yao et al., 2023). These contributions vividly illustrate why ecological perspectives matter: they enable researchers to capture learning and development as dynamic, situated, and historically embedded processes (Trickett and Rowe, 2012). By foregrounding the chronosystem and extended developmental trajectories, this line of work brings educational psychology closer to the core commitments of bioecological theory (Bronfenbrenner and Morris, 2006).

At the same time, the review suggests opportunities to further strengthen explanatory precision by explicitly theorizing how ecological conditions become developmentally consequential. The predominance of quantitative methods in the reviewed studies aligns with broader methodological trends in educational psychology, which have historically favored variable-centered, statistically driven approaches. However, the prevalence of SEM and multilevel modeling suggests that researchers are increasingly attending to the nested nature of educational data, which aligns conceptually with ecological perspective's recognition of multiple environmental levels. These methods were most often applied within cross-sectional designs to test mediation or moderation effects, rather than to model developmental processes unfolding over time. As a result, sophisticated analytical techniques were frequently used to map multilevel associations without fully engaging the temporal and processual dimensions central to bioecological theory. Moreover, greater attention to concepts such as proximal processes (Bronfenbrenner and Morris, 2006) could help connect rich narrative accounts with clearer mechanisms of change, enhancing the theoretical contribution of methodologically innovative studies.

### 7.4 Paradigm pluralism and theoretical integration

The diversity of ecological paradigms identified in this review (e.g., Bronfenbrenner's theory, Gibson's affordances, agency ecology, ecological validity) underscores the pluralistic nature of ecological thinking in educational psychology. As Bruineberg et al. (2024) argue, ecological psychology is better understood as a family of related approaches whose productive potential lies precisely in dialogue rather than unification. This diversity represents an important strength, reflecting the adaptability of ecological ideas across educational questions and contexts.

At the same time, comparative analyses reviewed earlier (Scarpa and Trickett, 2022; Tudge et al., 1996) highlight the importance of theoretical clarification when multiple frameworks are brought into dialogue. In the reviewed corpus, ecological theories were often combined with other perspectives to address complex psychological phenomena, demonstrating openness to integration. However, such integration often takes the form of juxtaposition. As shown in Section 6.4, multiple theories were frequently invoked to justify variable selection or interpret findings, but their underlying assumptions and potential complementarities were rarely examined.

Importantly, this pattern is not accidental. As scholars have long noted, ecological approaches have historically occupied a marginal position within mainstream psychology, where individualistic and representational traditions have dominated theory and method (Heft, 2013; Trickett and Rowe, 2012). One consequence of this marginalization is the tendency for ecological concepts to be adopted selectively or metaphorically, invoked to signal contextual sensitivity without sustained engagement with their relational and processual foundations. The juxtaposition of multiple ecological frameworks without fully integration observed in this review can be understood, at least in part, as an expression of this broader disciplinary history.

Future work could further strengthen this integrative potential by engaging more explicitly with the conceptual assumptions, complementarities, and boundaries among ecological traditions. For example, ecologically valid, real-time assessments could be used to examine proximal processes as they unfold within specific ecological systems, thereby linking methodological rigor of ecological validity with theoretical development of bioecological theory. Moreover, scholars could further explore dialogue between Chinese ecological philosophies (e.g., Daoist relational ontology and Confucian views of contextual moral cultivation) and Western ecological psychologies, particularly given that the majority of publications in this corpus were contributed by Chinese scholars (see Table 3). Such dialogue has the potential to enrich ecological paradigms in educational psychology by broadening its conceptual foundations and fostering culturally grounded interpretations of ecological relations.

### 7.5 Limitations of the review

Several limitations of this review should be acknowledged and addressed in future research.

First, the review was restricted to a single journal, *Frontiers in Psychology*. This focus was intentional, enabling in-depth analysis of one influential and interdisciplinary publication venue. However, ecological research published in other educational psychology journals may exhibit different patterns. The findings should therefore be interpreted as characterizing ecological framework use within this specific outlet.

Second, the search strategy relied on the explicit use of the terms *ecological, ecology*, or *ecosystem*. As a result, ecologically-oriented educational psychology research that does not employ this ecological terminology falls outside the scope of this review. For example, important studies engaging with related frameworks, such as sociocultural theory, activity theory, situated learning, Self-Regulated Learning (SRL) and Externally Regulated Learning (ERL), or cultural-historical psychology, may share ecological commitments without being captured by the search criteria. Moreover, as noted in Section 2, Gibson's ecological psychology, with its central emphasis on the concept of affordances (Gibson, 1979/2014), has been highly influential in educational research; however, studies drawing on affordance theory do not always explicitly identify their work as ecological in theoretical orientation. The boundaries of “ecological” thinking are theoretically contested, and alternative search strategies may yield a broader or differently constituted corpus. This represents a productive avenue for future systematic work.

Third, coding necessarily involved interpretive judgments. While a standardized protocol was employed and ambiguous cases were discussed among the research team to ensure consistency, the classification of studies into paradigmatic categories reflects the reviewers' understanding of ecological perspectives, which may differ from how original authors would characterize their work.

Despite these limitations, the review provides a systematic and theory-informed synthesis of ecological framework use within an important publication venue, offering a foundation for future cross-journal and cross-disciplinary comparisons.

## 8 Practical implications for educational practice and policy

Beyond its theoretical and methodological contributions, this review also yields implications for educational practice, policy, and intervention design. Although the reviewed studies were primarily research-oriented, the patterns identified point to recurring assumptions about where educational influence is located, and, by implication, where interventions are most likely to be targeted.

First, the strong empirical focus on microsystem-level factors, particularly family and school environments, suggests that much of the existing evidence base supports interventions targeting parenting practices, teacher–student relationships, and classroom climate. While such interventions are undoubtedly important, the relative neglect of mesosystem dynamics implies that coordination across settings remains an underdeveloped area of practice. The ecological perspective highlights that alignment between home and school expectations, communication, and support structures can be as consequential as the quality of any single environment (Bronfenbrenner, 1993). Practitioners and school leaders may therefore benefit from interventions explicitly designed to strengthen home–school linkages, rather than addressing family and school contexts in isolation.

Second, the emerging attention to exosystem and macrosystem factors has implications for policy design. Educational policies, institutional arrangements, and broader sociocultural conditions shape the proximal environments in which teaching and learning occur, yet these influences are rarely examined empirically. From an ecological perspective, policies that focus exclusively on individual performance or school-level accountability risk overlooking the structural conditions that enable or constrain educational development. Policymakers should therefore consider how policy decisions cascade through ecological systems to shape everyday educational experiences.

Third, the growing body of process-oriented and temporally sensitive research—particularly in studies of teacher emotions, agency, and resilience—suggests the value of supporting reflective, longitudinal professional development. Rather than treating teacher wellbeing or agency as static traits, educational practice can draw on ecological insights to recognize these as dynamic processes shaped by career stage, institutional reform, and broader sociocultural conditions. Practices such as sustained mentoring, collaborative inquiry, and narrative reflection align well with ecological perspective's emphasis on development over time and may be especially beneficial during periods of change or crisis.

Finally, the review highlights the importance of capacity building in ecological literacy among educational leaders and researchers. Clearer understanding of different ecological traditions—and of how they can be applied complementarily—can reduce the risk of superficial or symbolic adoption of ecological language.

## 9 Conclusion

This review shows that ecological paradigms have become a prominent feature of educational psychology research in *Frontiers in Psychology* (2013–2025). The contributions published in the journal provide not only a record of the ecological turn in educational psychology, but also a foundation for its continued theoretical and methodological evolution.

The findings show a clear evolution from initial, methodologically focused uses of ecological validity in the early years of the review period (2013–2017) toward a more theory-driven engagement with ecological systems perspectives, particularly Bronfenbrenner's framework, alongside emerging integrative approaches. At the same time, the review highlights variation in how ecological paradigms are operationalized, reflecting both the pluralistic nature of ecological thinking and the ongoing challenges of translating relational, dynamic, and process-oriented theories into empirical practice. By clarifying major ecological traditions, comparative perspectives, and functional patterns of paradigm use, the review contributes conceptual resources for more reflexive and theoretically informed engagement with ecological frameworks. In doing so, it underscores the value of pluralism, theoretical clarification, and methodological alignment in advancing ecological research in educational psychology.

Looking forward, the most promising direction is to deepen the ecological logic of inquiry: to examine mesosystem relations as interactional structures, to operationalize proximal processes with greater precision, and to develop theoretically and methodologically coherent integrations across ecological traditions (e.g., systems, affordances, ecological validity, and longitudinal methods). As educational environments continue to transform through technological change, global mobility, and societal disruption, ecologically grounded educational psychology will be increasingly necessary, not as a rhetorical gesture toward “context,” but as a disciplined framework for explaining how learning and development unfold in the real conditions of human lives.

## Data Availability

The original contributions presented in the study are included in the article/supplementary material, further inquiries can be directed to the corresponding author.
